# National multi-specialty robotic surgery training curriculum and implementation for UK surgical residents: a delphi consensus

**DOI:** 10.1007/s11701-026-03864-x

**Published:** 2026-08-29

**Authors:** Nader Francis, Taner Shakir, Esther McLarty, Fares Haddad, Adam Farquharson, Andrew Garnham, Somiah Siddiq, Aidan Bannon, Justin Collins, Nuha Yassin

**Affiliations:** 1 The Griffin Institute, Northwick Park and St Mark’s Hospital, Watford Road, London, HA1 3UJ UK; 2 Division of Surgery and Interventional Science, University College London, Charles Bell House, 43-45 Foley Street, London, W1W 7JN UK; 3 Plymouth Hospitals NHS Trust, Derriford Hospital, Plymouth, PL6 8DH UK; 4 University College London Hospitals NHS Foundation Trust, London, UK; 5 The Shrewsbury and Telford Hospital NHS Trust, Telford, UK; 6 Department of Surgery, Royal Wolverhampton Hospital, Wolverhampton Road, Wolverhampton, WV10 0QP UK; 7 Department of ENT, Head and Neck Unit, Heartlands Hospital, University Hospitals Birmingham NHS Foundation Trust, Birmingham, West Midlands UK; 8 Department of Surgery, Manchester University NHS Foundation Trust, Manchester, UK; 9 Department of Uro-Oncology, University College London Hospital, London, UK; 10 Department of Colorectal and General Surgery, University Hospitals Birmingham NHS Trust, Birmingham, West Midlands UK; 11 The Royal College of Surgeons of England, London, UK

**Keywords:** Robotic-assisted surgery, Surgical education, Training curriculum, Delphi consensus, Simulation, Competency-based training

## Abstract

Robotic-assisted surgery is now established across multiple surgical specialties in the United Kingdom, yet training for surgical residents remains variable, resource-dependent, and insufficiently standardised at national level. This study sought multispecialty expert consensus on the essential components of a national robotic surgery training curriculum for UK surgical residents. A four-round modified Delphi study was conducted between September and December 2025 under the RaDaR network of the Royal College of Surgeons of England. Round 1 used open-ended questionnaires with independent dual-coder thematic analysis; Round 2 was a hybrid face-to-face and online meeting with live anonymous electronic voting; Rounds 3 and 4 were online questionnaires addressing statements not yet reaching the pre-defined consensus threshold of 70% agreement or greater. The panel comprised consultant surgeons, surgical trainees, curriculum and training authorities, and industry representatives with deliberately bounded, non-clinical input. 25 participants completed the final round. Of 26 statements, 22 (84%) reached consensus. A three-tier framework of device, basic skills, and procedural training was endorsed, with device training introduced during Phase 1 (76%) and basic skills and procedural training introduced after Phase 1 (96% and 88% respectively). Competency assessment integrated within the Annual Review of Competence Progression was supported (84%), alongside multi-source funding; trainee self-funding was not endorsed. This consensus offers a pragmatic foundation for standardising robotic surgery training for UK residents, contingent on coordinated investment in platform access, simulation infrastructure, and faculty development.

**Supplementary Information:**

The online version contains supplementary material available at https://doi.org/10.1007/s11701-026-03864-x.

## Introduction

The integration of robotic-assisted surgery (RAS) into surgical practice has transformed operative approaches across multiple specialties. Over the past two decades, robotic platforms have evolved from emerging technology to established surgical tools, with increasing rates of adoption globally [1]. In the United Kingdom, robotic systems are now routinely utilised across urological, colorectal, and head and neck surgery, with expanding applications in thoracic surgery, hepatobiliary and vascular [2]. This rapid expansion has been highlighted by the Royal College of Surgeons of England (RCSEng) [3] and NHS National Cancer Plan [4], and with this comes an increasingly urgent need for structured, competency-based training programmes that can prepare the next generation of surgeons.

Despite this expansion, major barriers exist which limit equitable robotic training for surgical residents. High initial costs, limited operating time, and variable institutional expertise restrict trainee access, and can create geographical differences in training opportunities [2, 5, 6]. Although several robotic curricula and training frameworks have been proposed [7–13], no standardised national robotic surgery curriculum is integrated within UK surgical training pathways. Existing pathways vary considerably in content, assessment, and progression criteria [14, 15]. Consequently, there is heterogeneity, with reports suggesting that some trainees can complete training without any meaningful robotic experience [6]. This has downstream implications for career development, patient safety, and service sustainability. Attempting to address this gap is important, as emerging evidence suggests that structured robotic training programmes may support safer early operative performance within defined supervision frameworks [11, 16].

Several national and international organisations have sought to address this gap. The Royal College of Obstetricians and Gynaecologists offers a General Medical Council approved, optional special interest module in robotic surgery [17]. Internationally, examples including the Fundamentals of Robotic Surgery programme in North America [18], the European Society of Coloproctology (ESCP) robotic training guidelines [19], and the EAU Robotic Urology Section (ERUS) curriculum [11] have each demonstrated value. Additionally, The Royal College of Surgeons in Ireland (RCSI) has recently established Ireland’s first national soft-tissue robotic surgery pathway [12]. However, none of these programmes were designed for integration into the UK Intercollegiate Surgical Curriculum Programme (ISCP). Nor do they address the practical challenges of UK implementation, including regional variation in platform access, faculty availability, and protected training time. At present, only limited robotic assisted surgery competencies are formally mapped within the ISCP and the Annual Review of Competence Progression (ARCP) process. Within the UK, the ARCP is the annual panel review of each resident’s portfolio evidence against curriculum requirements, and determines progression to the next stage of training; it is therefore the mechanism through which any robotic competencies would be assured.

This study aimed to achieve multi-specialty expert consensus on the key elements of implementing a national robotic surgery training curriculum for UK surgical residents. Specifically, it sought to define the core curriculum structure, establish progression and assessment criteria, identify barriers and enablers to implementation, and provide recommendations for national adoption.

## Methods

### Study design

A four-round Delphi consensus study was conducted between September 2025 and December 2025. The modified approach incorporated a hybrid face-to-face meeting in Round 2 to facilitate dynamic discussion and real-time refinement of statements, whilst maintaining anonymous voting. Consensus was defined as agreement by 70% or more of respondents. This threshold was defined a priori by the steering committee, reflects the 70–80% range conventional in published Delphi methodology, and was selected to balance the need for meaningful agreement against the risk of exclusion across a deliberately heterogeneous multispecialty panel. The study was designed and reported in accordance with recognised recommendations for the conduct and reporting of Delphi studies (CREDES) [20].

### Participant recruitment

Participants were recruited to ensure comprehensive representation across surgical specialties, trainee representation, educational expertise, and stakeholder groups whilst maintaining a geographical spread of regions across the United Kingdom. Invitations were disseminated to the full membership of the RCS (Eng) RaDaR network, with links to centres across all four UK nations. Additional invitations were sent to robotic subspeciality societal members, those who provide strategic oversight of surgical training, and Associate Specialist (SAS) grade surgeons. Further expertise included Specialty Advisory Committee (SAC) members responsible for specialty-specific curriculum development and trainee assessment, Joint Committee on Surgical Training (JCST) representatives, robotic surgery platform industry partners who contribute to training programme development, and surgical training programme directors who oversee regional training delivery.

The aim was to assemble a panel broadly representative of specialties in which robotic platforms are established or rapidly emerging across the UK. However, final composition was shaped by which invited individuals were able and willing to participate. As a consequence, the panel represents an expert sample rather than a proportionate reflection of national surgical caseload. A complete list of the organisations invited and those ultimately represented is provided in Supplementary Table S1.

Industry representatives were also invited and contributed; their role was deliberately bounded to the domains of device training and logistical delivery, where platform-specific knowledge of system architecture and simulation infrastructure offered insight into what is technically available and feasible, rather than for clinical or educational content. Industry partners were granted a single vote per organisation and were excluded from voting on clinical competency standards, assessment frameworks, certification criteria, and funding governance.

### Delphi rounds

Round 1 comprised an online questionnaire distributed via a secure survey platform. The questionnaire comprised ten open-ended questions, presented in Table 1. The free-text response option allowed participants to elaborate on structured questions and introduce additional themes. Surgical participants provided demographic information including specialty, affiliations, role, robotic surgery experience (years and case volume), and geographical region. Invited participants provided expertise in device training and identified only one voting member, per industry partner, to be included in the Delphi responses.

**Table 1:** Round 1 questionnaire items

Item	Domain	Round 1 question
Q1	Curriculum components	What are the necessary components for a robotic training curriculum for surgical residents in the UK?
Q2	Implementation	How can these components be successfully implemented into the current structure of the multi-speciality as well as the individual training programmes?
Q3	Training stage	At what stage of surgical training should training in robotics be introduced for surgical residents?
Q4	Training stage	Which component of robotic training should be introduced at which stage of training?
Q5	Assessment	How should robotic training for surgical residents be assessed?
Q6	Certification	How can certification of competency in robotic training be measured?
Q7	Barriers	What are the perceived barriers for sustaining the delivery of robotic training to surgical residents?
Q8	Enablers	What are the perceived enablers for sustaining the delivery of robotic training to surgical residents?
Q9	Stakeholder support	What support can you provide to robotic training initiatives to surgical residents within your speciality?
Q10	Faculty support	What support is required for faculty / trainers to help with such initiative?

Open-ended questions upon which the Delphi exercise was based. Responses underwent independent dual-coder thematic analysis, from which the statements voted upon in Rounds 2 to 4 were derived. The full set of statements and response options used in the subsequent rounds is provided in Supplementary Table S2

Following completion of Round 1, qualitative responses underwent thematic analysis by the research team. Key themes were identified, coded, and synthesised to develop structured questions for Round 2. Areas of strong preliminary consensus were noted, whilst areas of disagreement were refined into specific propositions for further deliberation. Two researchers (NF and TS) independently coded free-text responses using an inductive thematic approach. Each response was assigned one or more thematic codes, which were grouped into categories. Any disagreements were resolved, where necessary, by a third researcher (NY). The coding framework was documented prospectively, with each consensus statement related to one or more Round 1 source themes. These were subsequently put to a vote in Round 2; no statement was introduced that did not originate in a Round 1 theme. Themes with broad preliminary agreement were converted into declarative statements presented for ratification, whereas themes in which responses diverged were reframed with defined response options. The resulting statement set constituted the Round 2 voting schedule.

Round 2 was conducted as a hybrid meeting at the Royal College of Surgeons of England on 18 September 2025, with simultaneous online participation via Microsoft Teams.

The session structure included:

- Introduction to the RCS device agnostic work, the need for curriculum standardisation and the gap in training needs
- Presentation of Round 1 thematic analysis results
- Multi-specialty panel discussion
- Live anonymous electronic voting on refined propositions using real-time polling software (Mentimeter, https://www.mentimeter.com)
- Facilitated discussion of voting results
- Iterative re-voting on modified propositions where necessary

This format enabled refinement of qualitative data and discussion whilst capturing quantitative consensus data. Participants could raise questions, challenge assumptions, and propose alternative formulations. Voting was anonymous to reduce hierarchical bias. Where discussion identified ambiguity or contested terminology within a statement, the wording was amended and the revised statement re-presented for voting within the same session; such amendments were confined to the phrasing of propositions already derived from Round 1 and did not introduce new domains. Of the Round 2 participants, 11 attended in person and 14 joined remotely via Microsoft Teams. To mitigate the risk that in-person attendees might dominate deliberation, all voting was conducted electronically and anonymously through the same real-time polling platform irrespective of attendance mode, ensuring each participant’s vote carried equal weight. The facilitator also actively invited contributions from online participants during discussion. Graphical representation of live responses were hidden to mitigate response bias.

Round 3 consisted of an online questionnaire distributed only to participants who had responded in Rounds 1 and 2. Round 3 presented only those statements that had not yet reached the pre-defined consensus threshold (≥ 70%) in Round 2. Statements achieving consensus at Round 2 were considered finalised, however, for ranking questions participants were asked to confirm acceptance of proposed rankings or suggest amendments. An additional Round 4 was distributed electronically in order to resolve remaining areas of disagreement.

### Data analysis

Qualitative data from open-ended responses were analysed thematically. Two researchers independently coded responses, with disagreements resolved through discussion. Key themes were identified, and illustrative quotations were selected to represent participant perspectives.

Quantitative data were analysed using descriptive statistics. For yes/no questions, the proportion agreeing was calculated. For ranking questions, participants ordered items by direct placement within the polling platform, which returned an aggregated group order rather than item-level distributions; this order was presented at the following round for endorsement. The percentages reported against ranked items therefore denote the proportion endorsing the aggregated order in full rather than the proportion selecting an individual item. Where no individual response option to an ordinal question reached the consensus threshold, adjacent categories were pooled post hoc for descriptive purposes; such pooled proportions are reported as descriptive only and were not treated as satisfying the consensus definition, which was applied to individual response options.

## Results

Across four rounds of the modified Delphi process, consensus was sought on 26 statements with 139 response options, of which 22 statements (84%) achieved the pre-defined consensus threshold of 70% or more agreement. All statements are presented in Supplementary Table S2. Twenty-five respondents completed the consensus process. The stakeholder representation and specialty distribution of participants are presented in Tables 2 and 3 respectively.

**Table 2:** Stakeholder representation of respondents (Round 3, n=25)

Stakeholder group	n	Percentage
Consultant surgeon	10	40%
Surgical trainee or SAS surgeon	5	20%
Industry representative	5	20%
Educationalist or training authority (SAC, JCST, or training programme director)	5	20%
Total	25	100%

Round 3 was circulated only to participants who had completed Rounds 1 and 2. Each respondent is assigned to a single stakeholder group. JCST, Joint Committee on Surgical Training; SAC, Specialty Advisory Committee; SAS, Specialty, Associate Specialist and Specialist grade

**Table 3:** Specialty distribution of respondents (Round 3, n=25)

Specialty	n	Percentage
Colorectal and General Surgery	5	20%
Urology	3	12%
Vascular Surgery	3	12%
Orthopaedic Surgery	3	12%
Thoracic Surgery	1	4%
Neurosurgery	1	4%
Head and Neck Surgery	1	4%
Oral and Maxillofacial Surgery (FDS)	1	4%
No clinical specialty recorded	7	28%

Round 3 was circulated only to participants who had completed Rounds 1 and 2. Percentages are calculated across all 25 respondents. A clinical specialty was not recorded for the five industry representatives or for two further respondents. FDS, Faculty of Dental Surgery

Consultant surgeons represented the largest single group with ten respondents (40%). Surgical trainees and SAS surgeons contributed five respondents (20%), educationalists and training authorities five (20%), and industry representatives five (20%), with the full distributions in Tables 2 and 3. Geographical representation spanned multiple regions of England, including the West Midlands, London, the North West, the South West, the East of England, and Yorkshire, together with representation from Northern Ireland. Invitations were extended to the full RCS (Eng) RaDaR membership; however, no participants from Scotland or Wales were able to attend.

The final participant group therefore comprised consultant surgeons with established robotic expertise across multiple specialties; surgical trainees; and curriculum and training authorities responsible for maintaining standards and assessment. The educationalists and training authorities recorded in Table 2 comprised individuals holding SAC, JCST, and training programme director roles, reflecting the bodies that would ultimately govern implementation of any curriculum within the ISCP and ARCP framework.

### Training framework definitions

Consensus was achieved on definitions for the three-tier training framework:

Device training was defined as: “Device training involves understanding the components of the platform, buttonology (including energy), docking and undocking, instruments and changes, emergency undocking” (88% consensus).

Basic skills training was defined as: “Basic skills training involves performing simulation training tasks (on VR, dry and wet lab), excluding surgical procedures” (76%).

Procedural training was defined as: “Procedural training involves performing either part or full surgical procedures within a modular training setting (including on VR, dry or wet lab)” (88%).

### Training stage progression

Consensus was achieved on the timing of introduction of device training during Phase 1 (Core Surgical Training years 1–2) (76%), with the optimal timing for introducing basic skills training to be after Phase 1 (96%). Consensus was also achieved for introducing the procedural aspect of training, with 88% stating it should be after Phase 1.

### Simulation requirements

The following simulator components achieved consensus as essential prerequisites prior to clinical console training:

- Basic system control (> 80%)
- Camera control (> 80%)
- Clutching (> 80%)
- Dissection (> 80%)
- Energy use (> 80%)
- Retraction (> 80%)
- Fourth arm control (where applicable) (78%)
- Suturing (78%)

Respondents noted that fourth arm control is not universally applicable across all specialties, with limited relevance in head and neck surgery and orthopaedics where three-arm configurations, and navigation-based platforms are standard practice. This may also apply to non-cavity work such as non-cavity vascular surgery and plastic surgery. More broadly, discussion during the consensus process recurrently distinguished cavitary robotic surgery from non-cavitary applications such as orthopaedic and spinal platforms. Participants observed that the latter function predominantly as navigation or decision support systems enhancing established procedures, with device training that is more software- and planning-oriented than instrument-handling oriented, and that the framework would need to accommodate this heterogeneity. The educational implications of this distinction are examined in the Discussion.

With respect to guidance on virtual reality competency, consensus opinions highlighted an 80% threshold (84%) on a guide for simulation benchmarks prior to progressing through the tiered training pathway. Factors that did not reach consensus were competency-based assessment focusing on holistic performance (12%), and trainer approval as the primary determinant (4%).

No single response option for the minimum number of simulator hours before progression reached the consensus threshold. When the adjacent 10–20 and 20–30 h categories were pooled post hoc, 77% of respondents favoured a requirement within the 10–30 h range; consistent with the aforementioned approach. This pooled proportion is descriptive and was not treated as satisfying the consensus definition. Notably, 22% emphasised that competency-based rather than time-based progression should be the primary driver. One participant observed that “40 poor hours is less valuable than 5 h done well”, highlighting concerns that arbitrary time-based requirements may incentivise box-ticking rather than genuine skill development.

### Simulation models

Consensus was not achieved on which specific simulation training models were best placed to be used at each aforementioned training stage. Respondents indicated varying preferences based on specialty requirements, resource availability, and educational objectives at different training stages.

For device training, no single model achieved consensus, with the most common selections being synthetic models (56%), low-fidelity hydrogel models (44%), and virtual reality simulators (44%).

For basic skills training, low-fidelity hydrogel models were the most common (67%). Other commonly selected models included synthetic models (56%), 3D printed models (52%), and high-fidelity hydrogel models (44%), though these did not reach the consensus threshold.

For procedural training, consensus was also not achieved on specific models. The most common preferences were ex vivo animal tissue (56%), cadaver models (56%), 3D printed anatomical models (52%), and high-fidelity hydrogel models (44%).

### Bedside assistant experience

A minimum number of 20 cases as bedside assistance was agreed on (77%) before progressing to console training.

### Competency assessment

The overall framework for competency assessment achieved strong consensus, with the existing ARCP process receiving 84% agreement. Assessment modalities and specific competence requirements for each phase of training were also explored (Tables 4 and 5). The ranked options presented to respondents included the Global Evaluative Assessment of Robotic Skills (GEARS), procedure-based assessments (PBAs), multiple consultant reports (MCRs), and multiple-choice question (MCQ) testing.

**Table 4:** Assessment modalities for each training phase

Training stage	Consensus	Assessment methods (Ranked Priority)
Device training assessment	84%	1. Simulation score (VR) 2. Console skills (GEARS) 3. Online test (MCQ) 4. Procedure-based assessments (PBAs) 5. Multiple consultant reports (MCR) 6. Video review 7. Logbook numbers
Basic skills training assessment	80%	1. Console skills (GEARS) 2. Simulation score (VR) 3. Procedure-based assessments (PBAs) 4. Online test (MCQ) 5. Multiple consultant reports (MCR) 6. Logbook numbers 7. Video review
Procedural training assessment	80%	1. Procedure-based assessments (PBAs) 2. Logbook numbers 3. Video review 4. Multiple consultant reports (MCR) 5. Console skills (GEARS) 6. Simulation score (VR) 7. Online test (MCQ)

Rank order was derived from participants’ direct ordering of items and confirmed at the subsequent round; the consensus percentage denotes the proportion endorsing the aggregated order in full. GEARS, Global Evaluative Assessment of Robotic Skills; MCQ, multiple-choice question; VR, virtual reality

**Table 5:** Certification components for each training phase

Training stage	Consensus	Certification components (Ranked priority)
Device training certification	88%	1. Simulation 2. Docking (“set-up” more inclusive) 3. Online Testing 4. Dry Lab 5. Wet Lab
Basic skills certification	96%	1. Procedural Workplace-Based Assessments 2. Objective Metrics (e.g. motion analysis) 3. Video Review 4. Supervised Cases
Procedural training certification	80%	1. Procedural Workplace-Based Assessments 2. Supervised Cases 3. Objective Metrics (e.g. motion analysis) 4. Logbook 5. Video Review 6. Clinical Outcomes

Rank order was derived as for Table 4

### Funding

The principle that Deanery and NHS funding should cover training costs achieved complete agreement (100%) in Round 2. Additionally, 84% of respondents agreed by Round 3 that industry should contribute to residents’ training funding. Local hospital funding also reached 80% consensus as an appropriate funding source. These represented three distinct funding-source statements rather than re-voting on a single proposition. The statement on Deanery and NHS funding reached consensus at Round 2 and was therefore finalised and not re-circulated. The separate statements regarding industry contribution and local hospital funding had not reached the consensus threshold at Round 2 and were accordingly carried forward to Round 3, consistent with the iterative process described in the Methods.

### Training delivery

**Fig. 1:**
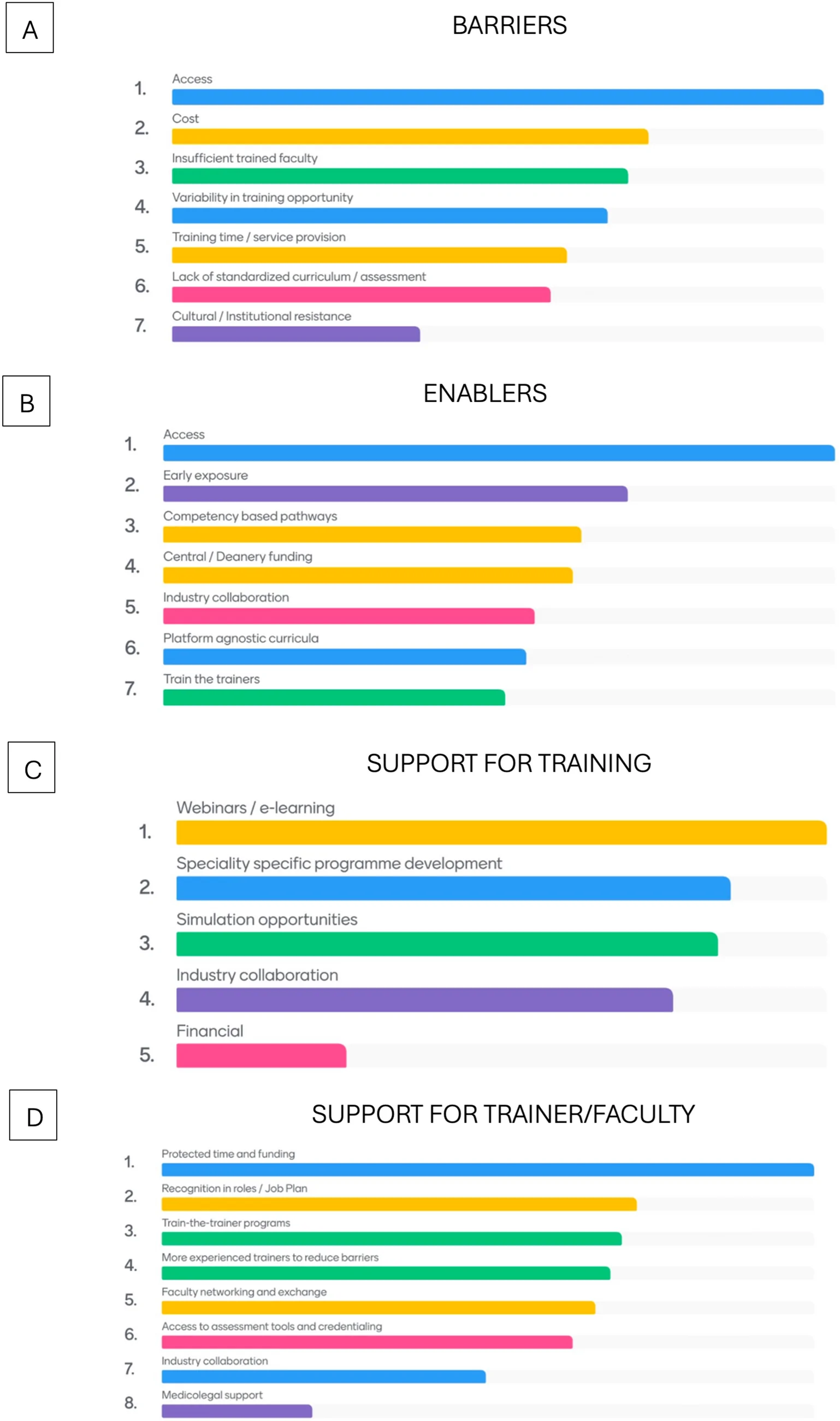
Factors Pertaining to Training Delivery with Percentage Consensus. Percentages denote the proportion of respondents endorsing the aggregated rank order for each domain. **A **Ranking of Perceived Barriers to Training Delivery (88%), **B. **Ranking of Perceived Enablers to Training Delivery (84%), **C **Ranking of the Support which could be provided for Training Initiatives (84%), **D **Ranking of Faculty/Trainer Support Requirements (92%)

**Fig. 2:**
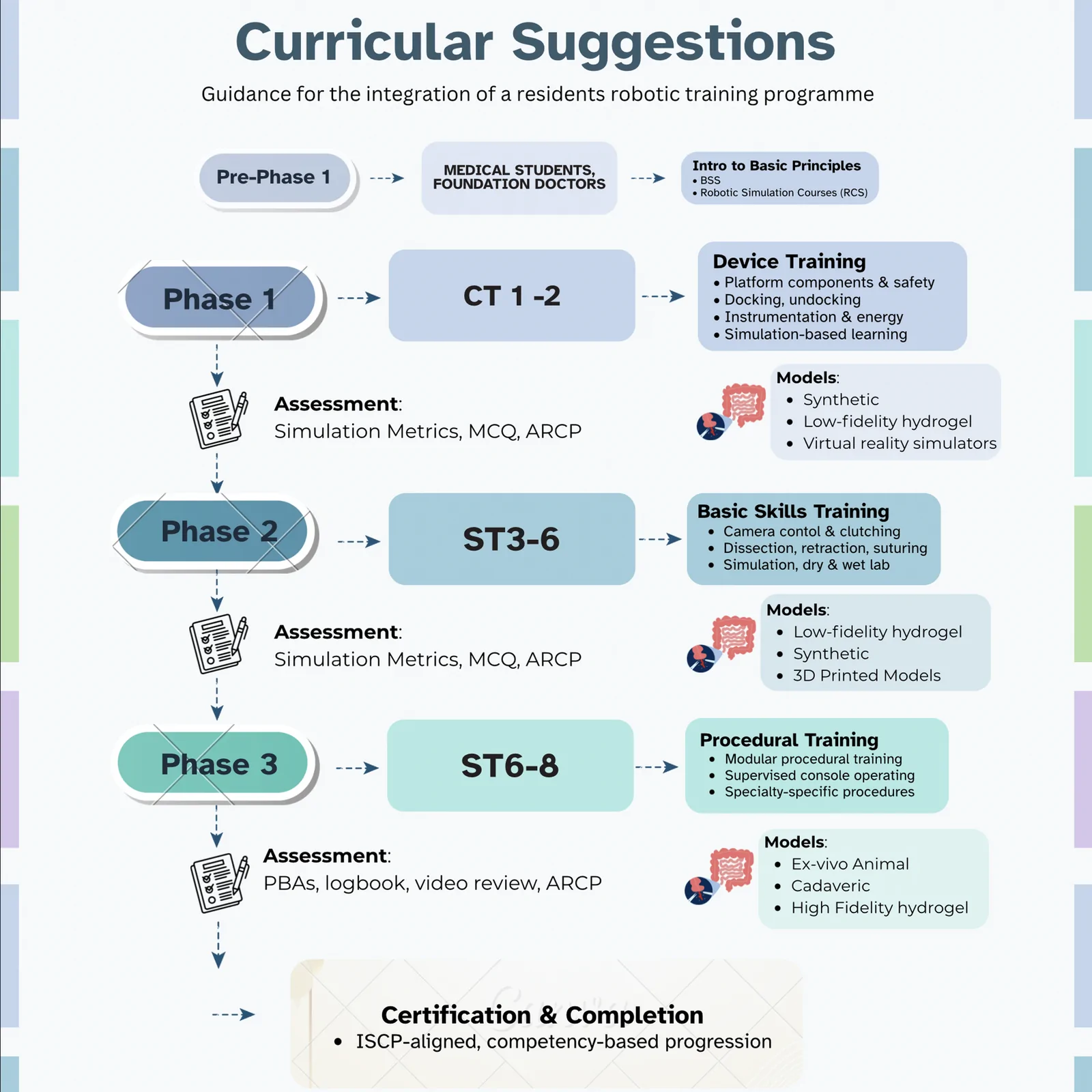
Suggestions for robotic curriculum implementation for surgical residents

Participants were asked to identify and rank barriers and enablers to training, types of support that specialty associations could provide, and the support requirements for faculty and trainers. Each factor, with respective ranking, which all reached consensus is highlighted in Fig. 1. Access to robotic platforms and simulation facilities was identified as both the principal barrier and key enabler for implementation of robotic training for surgical residents. Protected training time, formally recognised within consultant job planning, together with sustainable funding support and training the trainer programme were identified as the highest priorities for development of robotic faculty training and programme delivery. Drawing these consensus elements together, Fig. 2 summarises the proposed framework as an integrated, stepwise approach to curriculum implementation, mapping the three training tiers against progression points, assessment modalities, and the infrastructural and faculty prerequisites identified above.

## Discussion

This modified Delphi study achieved multispecialty consensus on 84% of statements, defining a national framework for the robotic training of UK surgical residents within existing ISCP and ARCP structures. Consensus was sought on curriculum structure, progression, assessment, and the barriers, enablers, and funding required for delivery. The framework offers a pragmatic response to current variability and inequity in robotic training while supporting standardised, competency-based progression. Its wider adoption may be supported by coordinated “Train-the-Trainer” programmes and the integration of emerging technologies [21, 22].

A three-tier structure (device, basic skills, then procedural training) was endorsed, mirroring established UK training models [15, 23, 24], with training introduced from Phase 1. Progression was felt to be best placed as competency based rather than purely time based, anchored to simulation benchmarks, 10–30 h of simulator practice, and around 20 bedside-assistant cases before console work. Consensus was not reached on specific simulation models, though preferences shifted with stage: synthetic and low-fidelity models for device and basic skills, progressing to high-fidelity or cadaveric models for procedural training. A move towards hydrogel models even in advanced training was noted for their ethical value and avoidance of the cultural sensitivities with certain animal tissues.

The recommendation to begin device-level exposure in Phase 1 warrants justification given constraints on cost and access. It does not imply substantial early console operating; rather, it endorses foundational learning, largely simulation and technology based. Early exposure may demystify the technology, inform career decisions, and build transferable competencies while consultants may remain on their own learning curves, with modular procedural training reserved for those who will use it.

Distinct assessment priorities emerged at each tier: simulation metrics for device training, global console skills (GEARS) for basic skills, and procedure-based assessments when procedural training. These aimed to reduce subjectivity in certification. Embedding these within the established ARCP process attracted strong support (84%), reinforcing a competency-based model consistent with current ISCP practice.

Access to platforms and simulation was identified as both the principal barrier and the key enabler; without it, equitable delivery cannot be achieved irrespective of curriculum quality. Cost ranked second, reflecting the high capital investment (£1–2.5 million per platform) and operational expenses [25]. The consensus supported collaborative, multi-source funding across: deaneries, the NHS, hospitals, industry, and training bodies; with trainees, colleges, and specialty associations not expected to contribute. Faculty development was a further critical enabler: as consultants are themselves early on the learning curve, “Train-the-Trainer” schemes will be needed to build teaching capacity while trainees gain exposure in parallel.

The statements that did not reach consensus concerned mainly the precise timing of phase introduction, specialty-specific pathway differences, and exact progression thresholds such as simulation hours. These reflect the difficulty of balancing educational ideals against practical constraints across diverse specialties, and are possibly the areas most in need of specialty-level refinement.

It is important to consider that a consensus of this kind cannot, and should not, override the distinct operative content of individual specialties. Demands of intracavitary colorectal or urological surgery differ significantly from navigation-based orthopaedic or spinal work. The value of this exercise lies instead in formulating a shared foundational architecture. Common tier definitions, a competency-based progression model, and agreed principles for assessment, access, and funding are a blueprint onto which specialties can map their own content. Subsequent specialty specific refinements are the intended next steps.

Non-cavitary robotics warrants specific consideration. In orthopaedic and spinal surgery, current platforms function largely as navigation and decision support systems that enhance established procedures rather than enabling new ones, while plastic surgery represents a further, less mature non-cavitary field [26]. The quantitative haptic and biomechanical feedback from these systems may improve our understanding when returning to conventional techniques. Furthermore, their device training is substantially software-based and will need to evolve as artificial intelligence contributes to planning and guidance. The framework is intended to accommodate this trajectory.

Situating the framework within the ISCP allows robotic competencies to be mapped onto the existing curriculum, rather than forming a parallel syllabus. The present statements are weighted towards technical, device-specific skills. As platforms evolve, generic and non-technical capabilities, such as non-technical skills [27, 28], may prove more important, and future iterations should include these.

When comparing existing curricula including the ESCP colorectal guideline [19], most recommendations rest on low or very low quality evidence, a limitation echoed here. They also reflect a history of industry-led training, whereby the present work deliberately differs. The inclusion of industry here was confined to a bounded, collaborative role. The overall distinction in this piece of work is one of scope. Rather than adding specialty depth, this framework provides a scaffold onto which such programmes can be mapped.

Moreover, when comparing this with recent international consensus work [29], this study adds UK-specific implementation detail mapped to the ISCP. Its principles nonetheless have wider relevance: systems facing comparable pressures of platform proliferation and heterogeneous trainee exposure could adapt the structure, progression milestones, and funding sources proposed here. International agreement on these principles would strengthen the evidence base and support mutual recognition of competencies across borders.

## Limitations

Several limitations warrant acknowledgement. The panel included multiple specialties and training grades, including consultants, trainers, and residents, however participant numbers were modest and the Round 3 sample (n=25) is small for a study of national scope. Trainee representation in particular was limited (n=5, 20%), which is notable for a curriculum intended to serve residents, and comparable trainee-focused Delphi work has achieved broader representation. The panel composition was shaped by the practical reality of who was able to participate, and consequently does not proportionately mirror national robotic caseload; high-volume specialties such as urology may be under-represented relative to procedural activity, while colorectal and general surgery predominated. The absence of gynaecology, despite an established GMC-approved robotic module in that specialty, limits the claim to comprehensive representation. Moreover, there is a risk of selection bias towards individuals already engaged in robotic surgery, who may hold more favourable views than the wider surgical community. These recommendations should therefore be interpreted as early expert consensus rather than definitive guidance. Furthermore, the hybrid format of Round 2 carries a risk that participants attending in person may influence discussion more than those joining remotely; while anonymous electronic voting and active facilitation of online contributions were used to mitigate this, it cannot be excluded.

Geographically, the panel was also predominantly England-based. Although invitations were circulated to the full RCS (Eng) RaDaR membership, which includes members based across the four nations, and to named individuals in Scotland and Wales, the final panel drew almost exclusively from centres in England, with the exception of representation from Northern Ireland. Surgical training is run separately in each of the four UK nations, each with its own curricula, funding, and oversight arrangements. The framework proposed here is deliberately generic, platform-agnostic, and specialty-agnostic, and none of its constituent elements is specific to a single nation; the claim to national scope should nonetheless be read with this composition in mind. Progression towards a genuinely UK-wide curriculum will require formal engagement with the Royal College of Surgeons of Edinburgh, the Royal College of Physicians and Surgeons of Glasgow, and the Royal College of Surgeons in Ireland, together with NHS Education for Scotland, Health Education and Improvement Wales, and the Northern Ireland Medical and Dental Training Agency, alongside the specialty associations active in each nation. Broadening the panel accordingly is a priority for the next phase of this work.

With respect to industry participation, this was governed by predefined role boundaries. Participants were only able to contribute to discussions relating to device training and logistical implementation, and were excluded from competency and certification decisions. It was considered their input is important when discussing the nuances of device training. It is also acknowledged that industry representation in Round 3 (n=5) matched that of trainees and educational representatives, and that much device-level expertise can in principle be provided by consultant surgeons practising robotic surgery. The rationale for retaining a bounded industry contribution was to capture accurate, platform-specific knowledge of what training infrastructure is currently available and feasible across multiple competing systems, rather than to confer influence over educational standards. Nonetheless, future iterations should recalibrate this balance, reducing industry to a strictly non-voting advisory capacity and ensuring that device-training content is led by surgeon educators, with industry input confined to technical accuracy.

Further methodological constraints also warrant note. Ranking data were captured as an aggregated group order rather than as item-level score distributions, so the strength of preference between adjacent items cannot be quantified and the percentages reported against ranked items reflect endorsement of the order as a whole. In addition, although amendments to statement wording during the Round 2 meeting were confined to propositions already derived from Round 1, a formal audit trail recording each individual amendment was not maintained. As an expert consensus study (Level V evidence), the findings should be considered hypothesis-generating and an important first phase in development of a national robotic curriculum. Prospective studies evaluating learning curves, assessment validity, patient outcomes, and cost-effectiveness under the proposed framework are required. Furthermore, implementation mechanisms relating to ARCP integration, accreditation standardisation, funding governance, and evolving technologies will require ongoing review and refinement. Finally, formal patient and public involvement was not undertaken at this consensus-development stage, which addressed postgraduate training structures rather than patient-facing interventions; patient perspectives will nonetheless be important in subsequent phases evaluating the relationship between training pathways, patient safety, and outcomes.

## Conclusions

This modified Delphi study establishes the first national multispecialty consensus framework to support development and implementation of robotic surgery training for UK surgical residents. The proposed three-tier curriculum, incorporating defined progression criteria and competency-based assessment, provides a practical response to current variability and inequity in robotic training. Successful implementation will require coordinated national investment in robotic access, simulation infrastructure, faculty development, and standardised assessment processes.

## Supplementary Information

Below is the link to the electronic supplementary material.Supplementary file1 (DOCX 26 KB)Supplementary file2 (DOCX 19 KB)

## Data Availability

No datasets were generated or analysed during the current study.
