# Supplementary material for: National multi-specialty robotic surgery training curriculum and implementation for UK surgical residents: a delphi consensus
Source: J Robot Surg. 2026 Aug 29;20(1):907. doi: 10.1007/s11701-026-03864-x (PMC13525944; doi:10.1007/s11701-026-03864-x)
Supplement: Supplementary file 1 [file 11701_2026_3864_MOESM1_ESM.docx]

**Supplementary Table S2
*Delphi questionnaire items and response options***

| Domain | Question | Response options |
| --- | --- | --- |
| Curriculum | What are the necessary components for a robotic training curriculum for surgical residents in England/The UK? | 1. Theoretical and clinical knowledge 2. Bedside assistance 3. Simulation-based training dry lab skills 4. Wet lab training; 5. Other 6. Mentorship with console time 7. Non-technical skills 8. None of the above |
| Definitions | Do you agree with the definition of Device Training?  *Device training involves understanding the components of the platform, buttonology (including energy), docking and undocking, instruments and changes, emergency undocking.* | Yes / No / Other |
| Definitions | Do you agree with the definition of Basic Skills Training?  *Basic skills training involves performing simulation training tasks (on VR, dry and wet lab), excluding surgical procedures.* | Yes / No / Other |
| Definitions | Do you agree with the definition of Procedural Training?  *Procedural training involves performing either part or full surgical procedures within a modular training setting (including VR, dry or wet lab).* | Yes / No / Other |
| Training stage | At what stage of surgical training should initial device training in robotics be introduced? | Phase 1 / Phase 2 / Phase 3 / Other |
| Training stage | At what stage of surgical training should | Phase 1 / Phase 2 / Phase 3 / Other |
| Training stage | At what stage of surgical training should procedural training in robotics be introduced? | Phase 1 / Phase 2 / Phase 3 / Other |
| Simulation | Which simulation tasks should be performed before progressing to console training? | 1. Camera control 2. Clutching 3. Dissection 4. Energy 5. Retraction 6. 4th arm control 7. Suturing 8. Not applicable (e.g. ENT/Orthopaedics) 9. Other |
| Simulation | What should be the minimum simulation score before progression to console training? | 1. 80% 2. >90% 3. 100% 4. Other |
| Simulation | What should be the minimum number of simulation hours before progression to console training? | 1. 0–10 2. 10–20 3. 20–30 4. 30–40 5. 40+ 6. Other |
| Models | Which model should be used during **device** training? | 1. Synthetic 2. Hydrogel (low fidelity) 3. Hydrogel (high fidelity) 4. 3D printed 5. Ex vivo animal tissue 6. Live animal 7. Cadaver 8. None of the above 9. Other |
| Models | Which model should be used during **basic skills** training? | 1. Synthetic 2. Hydrogel (low fidelity) 3. Hydrogel (high fidelity) 4. 3D printed 5. Ex vivo animal tissue 6. Live animal 7. Cadaver 8. None of the above 9. Other |
| Models | Which model should be used during **procedural** training? | 1. Synthetic 2. Hydrogel (low fidelity) 3. Hydrogel (high fidelity) 4. 3D printed 5. Ex vivo animal tissue 6. Live animal 7. Cadaver 8. None of the above 9. Other |
| Bedside training | What should be the minimum number of bedside assistant cases before progressing to clinical console training? | 1. <5 2. 5–10 3. 10–20 4. 20+ 5. Other |
| Assessment | Who should assess competency for robotic training? | 1. Existing ARCP process 2. Specialty-specific society 3. Royal College 4. International organisation 5. Industry 6. Other |
| Assessment | How should device training be assessed? | 1. Simulation score (VR) 2. Console skills (GEARS) 3. Online test (MCQ) 4. Procedure-based assessments (PBA) 5. Multiple consultant report (MCR) 6. Video review 7. Logbook numbers |
| Assessment | How should basic skills training be assessed? | 1. Console skills (GEARS) 2. Simulation score (VR) 3. Procedure-based assessments (PBA) 4. Online test (MCQ) 5. Multiple consultant report (MCR) 6. Logbook numbers 7. Video review |
| Assessment | How should Procedural Training be assessed? | 1. Procedure-based assessments (PBA) 2. Logbook numbers 3. Video review 4. Multiple consultant report (MCR) 5. Console skills (GEARS) 6. Simulation score (VR) 7. Online test (MCQ) |
| Certification | How should device training be certified? | 1. Simulation 2. Docking 3. Online testing 4. Dry lab 5. Wet lab |
| Certification | How should basic skills training be certified? | 1. Procedural WBA 2. Objective metrics (e.g. motion analysis) 3. Video review 4. Supervised cases |
| Certification | How should advanced/procedural training be certified? | 1. Procedural WBA 2. Supervised cases 3. Objective metrics 4. Logbook 5. Video review 6. Clinical outcomes |
| Funding | In addition to Deanery/NHS, should anyone else cover the cost of robotic surgery training? | 1. Local hospital 2. Trainees 3. Industry 4. Other |
| Sustainability | What are the perceived barriers to sustaining robotic training? | 1. Access 2. Cost 3. Insufficient trained faculty 4. Variability in training opportunity 5. Training time/service provision 6. Lack of standardised curriculum/assessment 7. Cultural/institutional resistance |
| Sustainability | What are the perceived enablers for sustaining robotic training? | 1. Access 2. Early exposure 3. Competency-based pathways 4. Central/Deanery funding 5. Industry collaboration 6. Platform-agnostic curricula 7. Train the trainers |
| Stakeholder support | What support can you provide to robotic training initiatives within your specialty? | 1. Webinars/e-learning 2. Specialty-specific programme development 3. Simulation opportunities 4. Industry collaboration 5. Financial support |
| Faculty support | What support is required for faculty/trainers to help with such initiatives? | 1. Protected time and funding 2. Recognition in roles/job plan 3. Train-the-trainer programmes 4. More experienced trainers 5. Faculty networking and exchange 6. Access to assessment tools and credentialing 7. Industry collaboration 8. Medicolegal support |
