# Supplementary material for: National multi-specialty robotic surgery training curriculum and implementation for UK surgical residents: a delphi consensus
Source: J Robot Surg. 2026 Aug 29;20(1):907. doi: 10.1007/s11701-026-03864-x (PMC13525944; doi:10.1007/s11701-026-03864-x)
Supplement: Supplementary file 2 [file 11701_2026_3864_MOESM2_ESM.docx]

**Supplementary Table S1
*Invitees to Delphi Consensus Exercise***

| **First name** | **Surname** | **Organisation** | **Invitation outcome** |
| --- | --- | --- | --- |
| **Educationalists** | | | |
| Ginny | Bowbrick | Head of School of Surgery, NHS England | Attended |
| Justin | Collins | University College London | Attended |
| Andrew | Garnham | Confederation of Postgraduate Schools of Surgery (CoPSS) | Attended |
| Rebecca | Martin | Royal College of Surgeons of England Learning | Attended |
| Esther | McLarty | Joint Committee on Surgical Training (JCST) | Attended |
| Fares | Haddad | University College London | Declined |
| Paul | Sadler | Health Education England (HEE) | Declined |
| Gill | Tierney | General Surgery SAC | Declined |
| **RCS RaDaR Group** | | | |
| Francisca | Ferreira | University of Cambridge | Attended |
| Shakil | Ahmed | Liverpool University Hospitals NHS Foundation Trust | Attended |
| Leanne | Ashrafian | Guy's and St Thomas' NHS Foundation Trust | Attended |
| Ben | Challacombe | Guy's and St Thomas' NHS Foundation Trust | Attended |
| Adam | Farquharson | Shrewsbury and Telford Hospital NHS Trust | Attended |
| Nader | Francis | The Griffin Institute | Attended |
| Niall | McGonigle | Belfast Health and Social Care Trust | Attended |
| Sadasivam | Selvakumar | East and North Hertfordshire NHS Trust | Attended |
| Taner | Shakir | University College London | Attended |
| Vinita | Shekar | Royal College of Surgeons of England | Attended |
| Somiah | Siddiq | University Hospitals Birmingham NHS Foundation Trust | Attended |
| Kaji | Sritharan | York and Scarborough Teaching Hospitals NHS Foundation Trust | Attended |
| Nuha | Yassin | Royal College of Surgeons of England | Attended |
| Keith | Allison | Royal College of Surgeons of England | Declined |
| Jonathan | Barry | Swansea Bay University Health Board (Morriston Hospital) | Declined |
| Jonathan | Epstein | Royal College of Surgeons of England | Declined |
| Jonathan | Ghosh | Manchester University NHS Foundation Trust | Declined |
| Will | Hellier | Royal College of Surgeons of England | Declined |
| Ian | Kamaly-Asl | Royal College of Surgeons of England | Declined |
| Sam | Lewis | Royal College of Surgeons of England | Declined |
| John | McGrath | North Bristol NHS Trust | Declined |
| Vin | Paleri | The Royal Marsden NHS Foundation Trust | Declined |
| Francesca | Ruccia | The Royal Marsden NHS Foundation Trust | Declined |
| Rana | Sayeed | Royal College of Surgeons of England | Declined |
| Robert | Sayers | Royal College of Surgeons of England | Declined |
| Shailinder | Singh | Royal College of Surgeons of England | Declined |
| Naeem | Soomro | Royal College of Surgeons of England | Declined |
| Ian | Sugarman | Royal College of Surgeons of England | Declined |
| Hiro | Tanaka | Royal College of Surgeons of England | Declined |
| Jared | Torkington | University Hospital of Wales, Cardiff | Declined |
| Sujatha | Udayasankar | Royal College of Surgeons of England | Declined |
| Bhavin | Visavadia | Royal College of Surgeons of England | Declined |
| **Trainees** | | | |
| Sas | Banerjee | Royal College of Surgeons of England SAS community | Attended |
| Aidan | Bannon | Association of Surgeons in Training (ASiT) | Attended |
| Matthew | Harris | Association of Surgeons in Training (ASiT) | Attended |
| Ibrahim | Ibrahim | British Orthopaedic Trainees Association (BOTA) | Attended |
| George | Ninkovic-Hall | Liverpool University Hospitals NHS Foundation Trust | Attended |
| Raiyyan | Aftab | Association of Surgeons in Training (ASiT) | Declined |
| Oliver | Townsend | British Orthopaedic Trainees Association (BOTA) | Declined |
| **Industry** | | | |
| Richard | Bastier | Intuitive Surgical | Attended |
| Diego | Bresciani | Medacta | Attended |
| Louise | Frame | Stryker | Attended (non-voting) |
| Mariachiara | Guffanti | Medacta | Attended (non-voting) |
| Mike | Hope | Stryker | Attended |
| Daniel | Jones | Stryker | Attended (non-voting) |
| Elizabeth | Jones | Medtronic | Attended (non-voting) |
| Sara | Lazzaretti | Asensus Surgical | Attended |
| Bart | Pattyson | Johnson & Johnson | Attended (non-voting) |
| Robin | Thomas | MicroPort | Attended (non-voting) |
| Katherine | Winger | CMR Surgical | Attended |
| Vincent | Beysang | MicroPort | Declined |
| Gillian | Duncan | Intuitive Surgical | Declined |
| Karen | Felsmann | Karl Storz | Declined |
| Karen | Hughes | Stryker | Declined |
| Rick | Lombardi | Johnson & Johnson | Declined |
| Angela | Meyburg | Intuitive Surgical | Declined |
| Amanda | Quinn | Stryker | Declined |

*Named invitations are listed above. In addition, the invitation to participate was circulated to the full membership of the Royal College of Surgeons of England Robotic and Digital Assisted Surgery (RaDaR) group, which includes members based in Scotland and Wales; the table therefore records named invitees rather than the total reach of the invitation. Invitation outcome denotes whether an invitee took part in the Delphi process; “Declined” includes both explicit declines and invitations to which no response was received. Industry attendees marked non-voting are those beyond the single voting representative permitted for each organisation. Attendance denotes participation in the consensus exercise as a whole; 25 participants completed Round 3 and form the denominator for Tables 2 and 3 of the main manuscript. Some authors and collaborators contributed to the development of the manuscript but were unable to attend the consensus session.*
